# Supplementary material for: The rostroventral part of the thalamic reticular nucleus modulates fear extinction
Source: Nat Commun. 2019 Oct 11;10:4637. doi: 10.1038/s41467-019-12496-9 (PMC6789150; doi:10.1038/s41467-019-12496-9)
Supplement: Supplementary file 1 — Supplementary Information [file 41467_2019_12496_MOESM1_ESM.pdf]

Supplementary Information

The rostroventral part of the thalamic reticular nucleus modulates fear extinction

Lee et al., *Nature Communications*.

Supplementary Figures

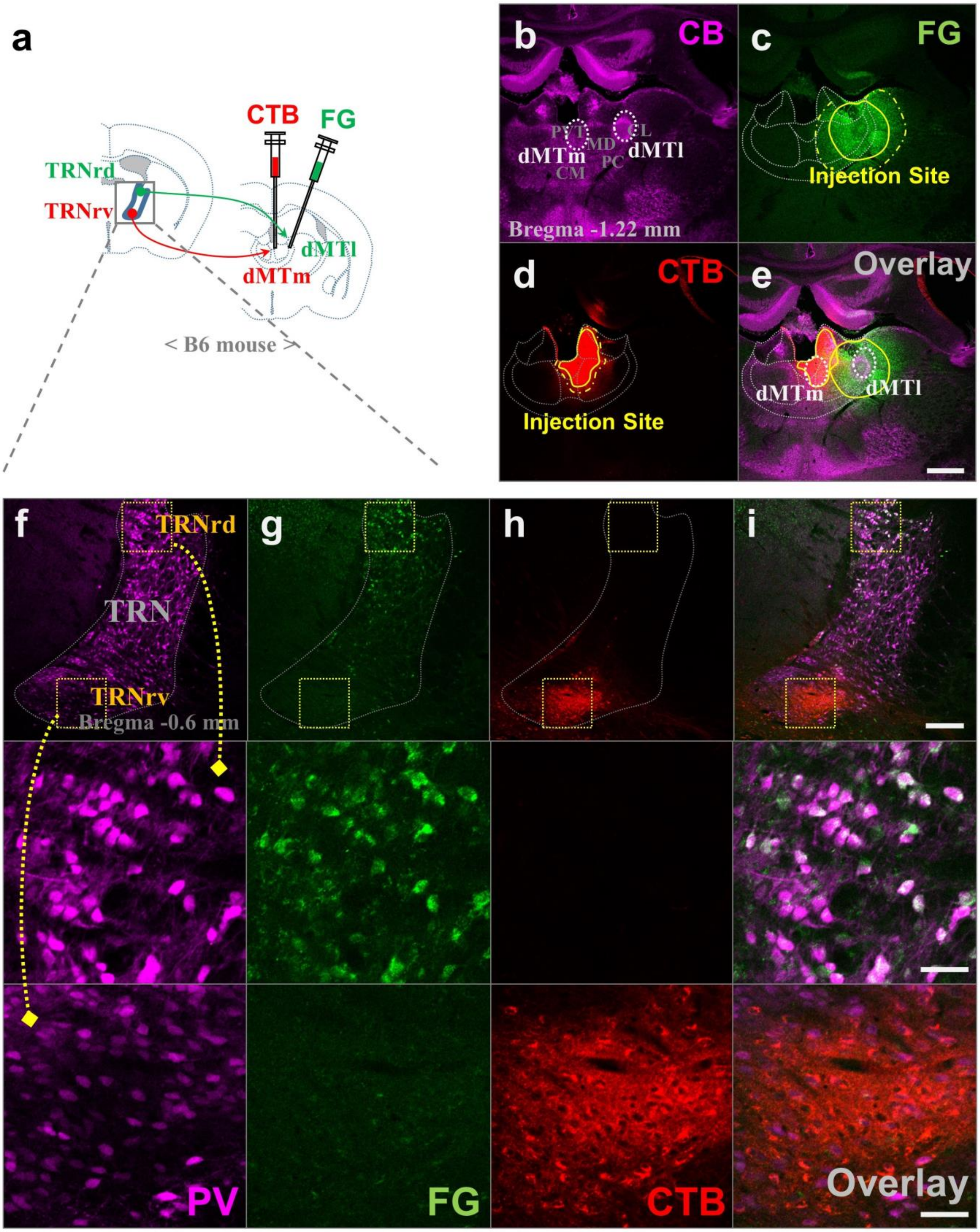
a
CTB
FG
TRNrd
TRNrv
dMTl
dMTm
< B6 mouse >
b
CB
PVT
CL
dMTm
MD
dMTl
CM
PC
Bregma -1.22 mm
c
FG
Injection Site
d
CTB
Injection Site
e
Overlay
dMTm
dMTl
f
TRNrd
TRN
TRNrv
Bregma -0.6 mm
g
h
i
PV
FG
CTB
Overlay

Supplementary Figure 1. Distinct projection patterns of the TRNrv and the TRNrd. In this case, the injection sites of two retrograde tracers were switched compared to Fig. 1. Related to Fig. 1.
a Schematic depiction of injections of retrograde tracers. **b** CB immunostaining delineates the boundaries of the limbic thalamus. **c** FG injection site. Solid and dotted yellow lines indicate the sites where the strong and the weak FG signals were observed, respectively. **d** CTB injection site. Solid and dotted yellow lines indicate the sites where the strong and the weak CTB signals were observed, respectively. **e** Overlaid image. Scale bar, 500 μm. **f** PV immunostaining delineates the boundaries of the TRN. Magnified images are shown in lower panels. **g** FG signals are observed only in the TRNrd but not in the TRNrv. **h** CTB signals are observed only in the TRNrv but not in the TRNrd . **i** Overlaid image. Scale bar, 200 μm. Magnified images. Scale bar, 50 μm.

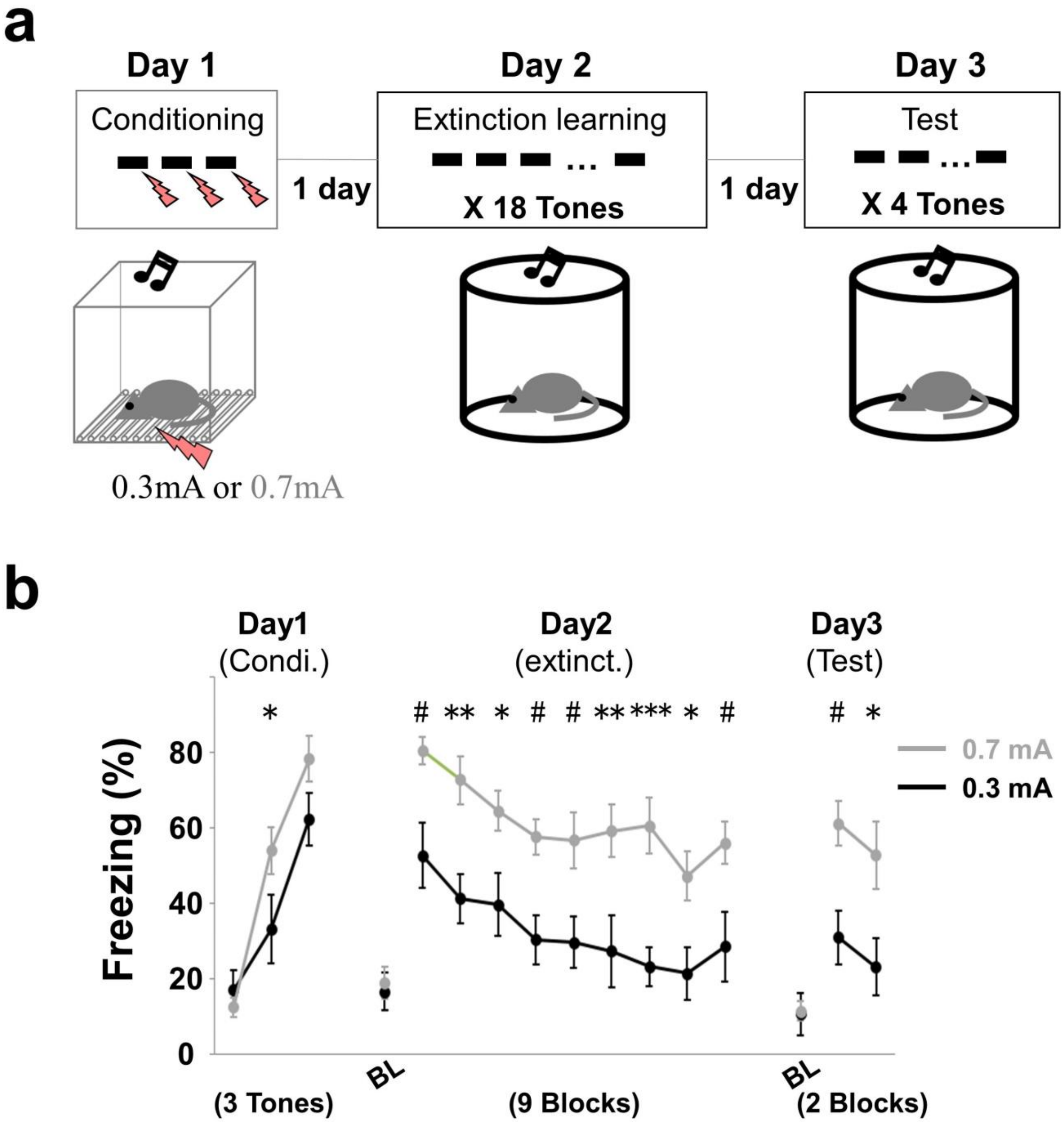

Supplementary Figure 2. The effect of different shock intensity in fear conditioning to the freezing levels during extinction learning and retrieval test. Related to Fig. 2.

a Schematic depiction for experimental protocol. During fear conditioning (Day 1), the mice were shocked with either the 0.3 mA shock or the 0.7 mA shock. **b** The 0.7 mA group showed significantly increased freezing levels than 0.3 mA group throughout the all blocks in extinction learning and retrieval test (n = 9 for 0.3mA group, n = 10 for 0.7mA group. 1st-9th blocks of Extinct., two-way RM ANOVA, $F_{(1,17)} = 16.815$, $P < 0.001$, 2 blocks of the test day, two-way RM ANOVA, $F_{(1,17)} = 9.654$, $P = 0.006$). No significant difference of the baseline freezing (BL, 30 sec before the first tone) between the groups was observed (n = 9 for 0.3mA group, n = 10 for 0.7mA group; baseline of Extinct. Two-tailed t-test, $t_{(17)} = -0.365$, $P = 0.719$; baseline of the test day. Mann-Whitney Rank Sum Test, U = 28.5, P = 0. 19). One block is the average of two tone trials. All

data are presented as mean ± SEM. *P < 0.05, #P < 0.01, **P < 0.005, ***P < 0.001. See Supplementary Table 1 for values of post hoc test.

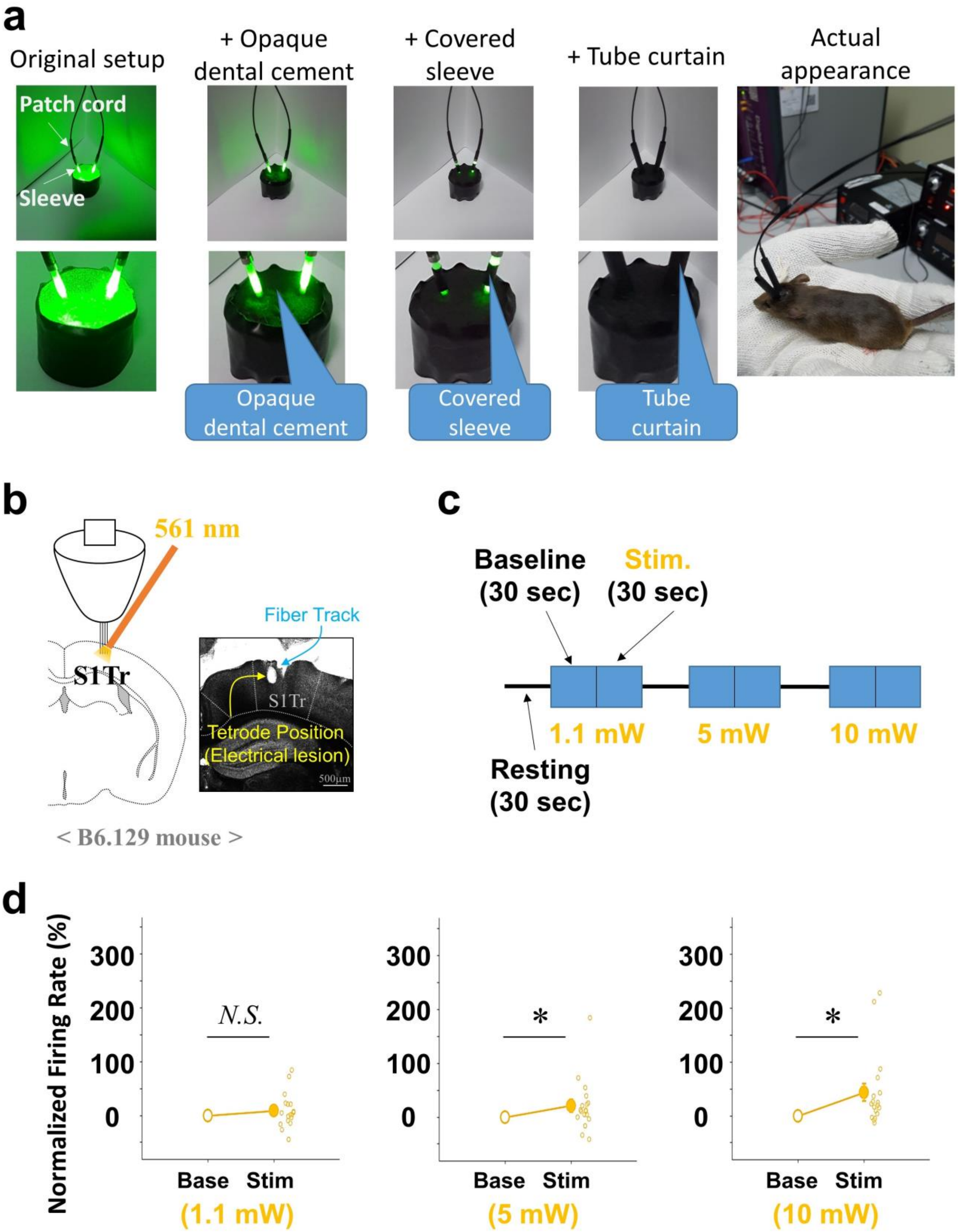
a
Original setup
+ Opaque dental cement
+ Covered sleeve
+ Tube curtain
Actual appearance
Patch cord
Sleeve
Opaque dental cement
Covered sleeve
Tube curtain
b
561 nm
S1Tr
Fiber Track
S1Tr
Tetrode Position (Electrical lesion)
500μm
< B6.129 mouse >
c
Baseline (30 sec)
Stim. (30 sec)
1.1 mW
5 mW
10 mW
Resting (30 sec)
d
Normalized Firing Rate (%)
300
200
100
0
N.S.
*
*
Base Stim
(1.1 mW)
(5 mW)
(10 mW)

Supplementary Figure 3. The experimental setup for optogenetic experiment. Related to Fig. 2, 4 and 5.
a Comparison between original optogenetic setup and our optogenetic setup. By using opaque dental cement, covered sleeve and tube curtain, the light leakage was completely blocked. **b** Schematic depiction for the recording. To examine heating effect, the changes of firing rates to different intensities of light power were measured. The tetrode and optical fiber were implanted into the trunk region of primary somatosensory cortex (S1Tr). **c** Recording protocol. Single units were recorded while each intensity (1.1mW, 5mW and 10mW) of light (561 nm) was applied. The recording was performed in home cage. **d** The firing change was not observed by the light stimulation with 1.1 mW (= 140 mW/mm^2 with 100μm core fiber) intensity but observed by 5 mW (= 637 mW/mm^2) and 10 mW (= 1274 mW/mm^2) intensities (n=18 neurons, 3 mice; 1mW, Two-tailed One-Sample t-test, $p = 0.233$; 5mW, One-Sample Signed Rank Test, $W = 103.0$, $P = 0.024$; 10mW, One-Sample Signed Rank Test, $W = 91.0$, $P = 0.048$). All data are presented as mean ± SEM. N.S., not significant. *$P < 0.05$.

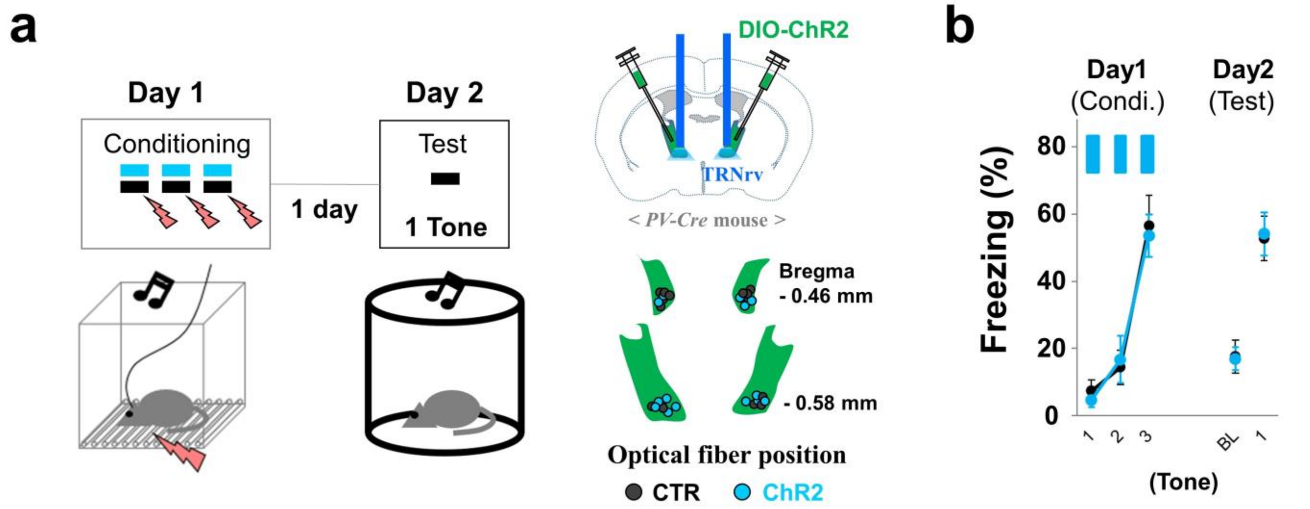

Supplementary Figure 4. The effect of optogenetic stimulation of the TRNrv during fear conditioning. Related to Fig. 2.
a Left, Schematic depiction for optogenetic excitation of the TRNrv during fear conditioning. Right, The positions of fiber tips are marked by blue dots for stimulated group and gray dots for control group. **b** The optogenetic stimulation of the TRNrv during fear conditioning did not change the freezing levels during the conditioning (Day 1, Condi., two-way RM ANOVA, $F_{(1,12)} = 0.0387$, $P < 0.847$) or the retrieval test (Day 2, Test, two-tailed t-test, $t_{(12)} = 0.902$). All data are presented as mean ± SEM. N.S., not significant.

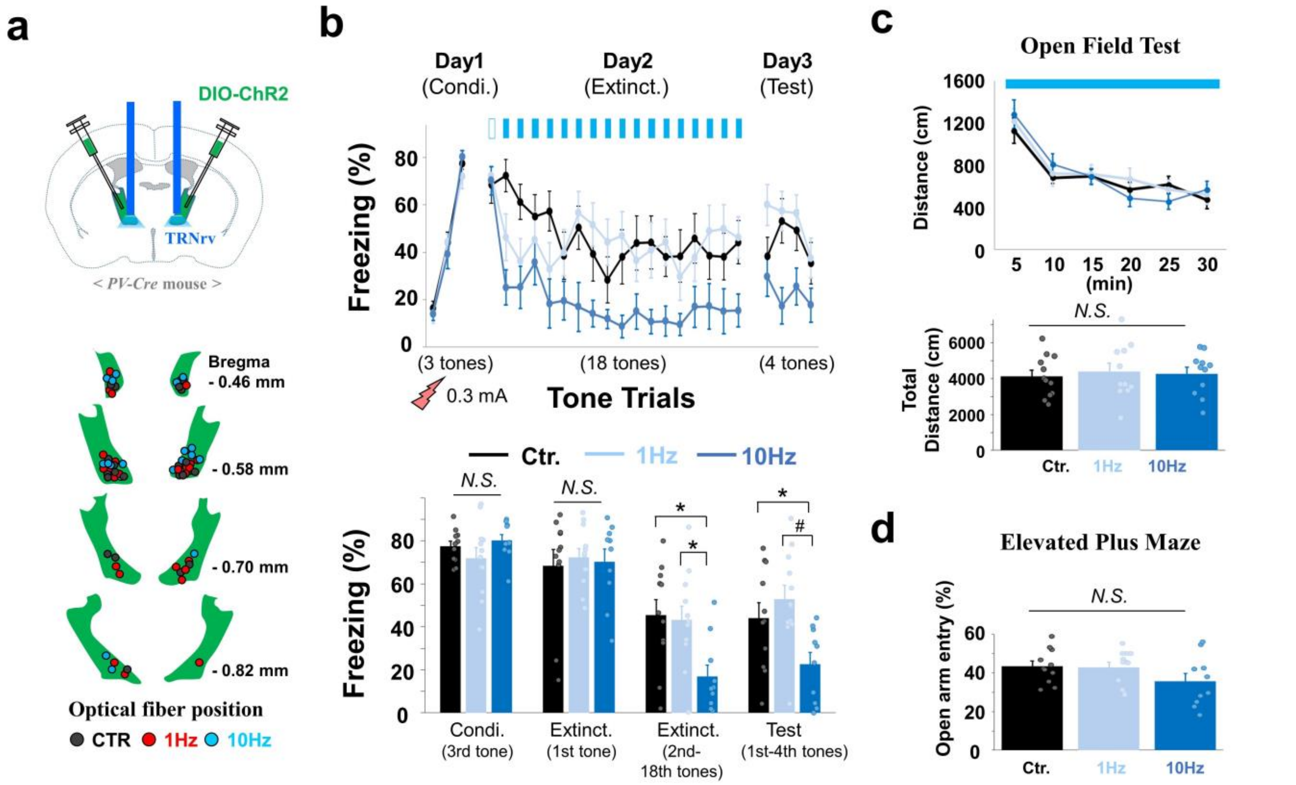

Supplementary Figure 5. The effect of 1Hz or 10Hz optogenetic stimulation of the TRNrv on fear extinction, anxiety level and locomotor activity. Related to Fig. 2.

a Schematic depiction for optogenetic excitation of the TRNrv. The positions of fiber tips are marked by blue dots for 10Hz stimulated group, red dots for 1Hz stimulated group and gray dots for control group. **b** Top panel, Behavioral result shown in Fig. 2d is replicated in a different facility. Note that overall freezing level of the control group is higher compared to Fig. 2f and 2h, which may be due to the environmental difference between the facilities. Consistent with Fig. 2d, 10 Hz optogenetic excitation of the TRNrv during extinction learning induced decreased freezing levels during extinction learning and during retrieval test whereas 1Hz stimulation did not affect (n = 11 for control, n = 12 for 1Hz stimulated group, n=10 for 10Hz stimulated group; 2nd–18th tones of Extinct., two-way RM ANOVA, $F_{(2,30)} = 5.772$, P = 0.008, Holm-Sidak method, (Ctr) vs. (10Hz), t = 3.062, P = 0.014, (Ctr) vs. (1Hz), t = 0.250, P = 0.804, (10Hz) vs. (1Hz), t = 2.881, P = 0.014; four tones of the test day, two-way RM ANOVA followed, $F_{(1, 23)} = 5.623$, P = 0.008, Student-Newman-Keuls Method, (Ctr) vs. (10Hz), q = 3.234, P = 0.03, (Ctr) vs. (1Hz), q = 1.395, P = 0.332, (10Hz) vs. (1Hz), q = 4.660, P = 0.007). Bottom panel, the quantification of the data in top panel. Condi., conditioning. Extinct., extinction. **c** No significant difference was observed among the control group (n = 12), the 1Hz group (n = 11) and the 10Hz group (n = 11) in locomotor activity (two-way RM ANOVA, $F_{(2,31)} = 0.118$, P = 0.889). **d** No significant difference was observed among the control group (n = 11), the 1Hz group (n = 12) and the 10Hz group (n = 10) in anxiety level (one-way ANOVA, $F_{(2,30)} = 1.816$, P = 0.180). All data are presented as mean ± SEM. N.S., not significant. *P < 0.05, #P < 0.01. See Supplementary Table 1 for values of post hoc test.

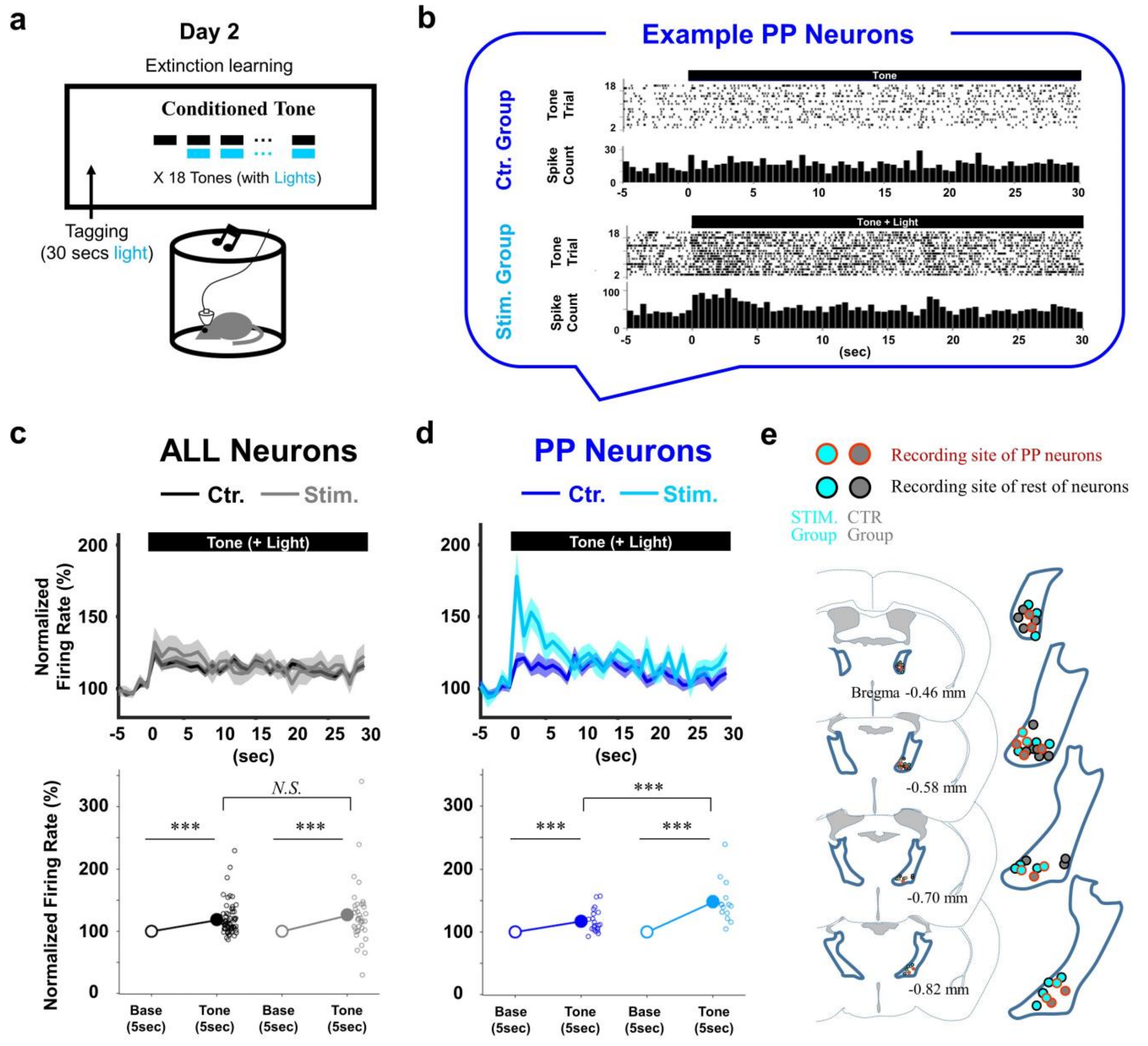

Supplementary Figure 6. Spiking correlates of TRNrv neurons in the facilitation of fear extinction. Related to Fig. 3. **a** Experimental protocol. During the extinction learning, 10Hz light stimulations were delivered with the tones to stimulated group but not control group. **b** Example unit response of PP neurons to conditioned tones without light stimulation (control group, upper row) and with light stimulation (stimulated group, lower row). **c** Top, Normalized firing responses of ALL neurons to conditioned tones without light stimulation (black) or with light stimulation (gray). Bottom, Responses of 5 sec baseline and first 5 secs of the tone are compared. The light stimulation did not induce significant difference in firing rates between the groups in the analysis of ALL neurons (between the groups in low panel, n = 65 neurons, 8 mice for control group, n = 38, 9 mice for stimulated group, Mann-Whitney rank sum test, U = 1080.000, P = 0.291). In each group, significant increase of firing rate to conditioned tones were observed (Control group, black line in low panel, one-sample signed rank test, Z = 5.944, P < 0.001; Stimulated group, gray line in low panel, one-sample signed rank test, Z = 3.633, P < 0.001). **d** Top, Normalized firing responses of PP neurons to conditioned tones without light stimulation (dark blue) or with light stimulation (bright blue). Bottom, Responses of 5 sec baseline and first 5 secs of the tone are compared. The light stimulation induced significant difference in firing pattern between the groups in the analysis of PP neurons (n = 21 neurons, 5 mice for control group, n = 14, 6 mice for stimulated group, Mann-Whitney rank sum test, U = 52.000, P = 0.001). In each group, significant increase of firing rate to conditioned tones were observed (Control group, black line in low panel, one-

sample signed rank test, Z = 3.632, P < 0.001; Stimulated group, gray line in low panel, one-sample signed rank test, Z = 3.296, P < 0.001). **e** Electrode positions. Recording sites of PP neurons are marked by red circle. Recording sites of stimulated group and control group are filled with cyan and gray colors, respectively. All data are presented as mean ± SEM. *N.S.*, not significant. $^{***}P <= 0.001$.

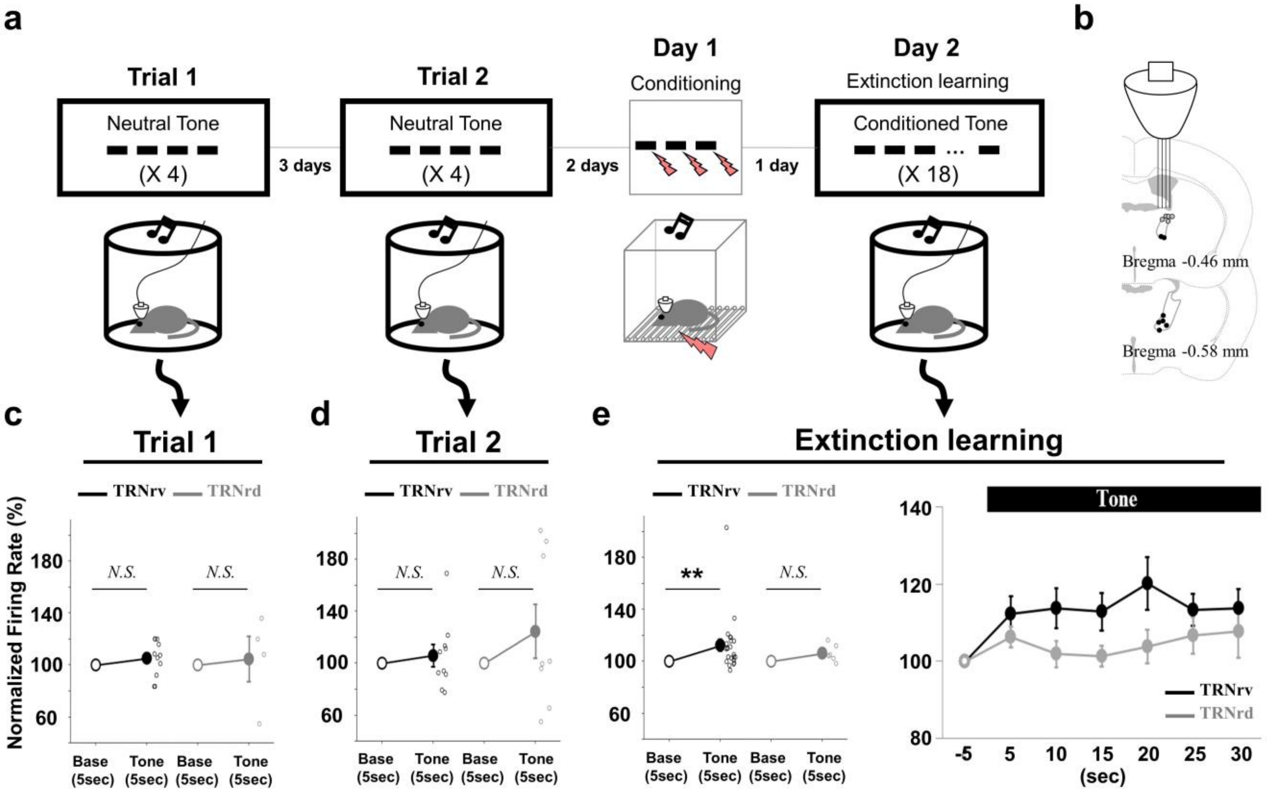

Supplementary Figure 7. Repeated exposure of neutral tones does not induce significant responses of TRNrv neurons to the neutral tones. TRNrd neurons do not show significant responses to conditioned tones. Related to Fig. 3.
a Experimental protocol. The mice were exposed to the neutral tones with 3 days interval, which is equivalent to the interval between neutral tone test and conditioned tone test (Fig. 3), and then exposed to conditioned tones. **b** Recording site were marked by empty (TRNrd recording) or filled (TRNrv recording) circles. **c, d** Responses of 5 sec baseline and first 5 secs of the neutral tone are compared. Presentation of neutral tones (**c**) or re-exposure of neutral tones (**d**) did not induce significant responses either in TRNrv neurons (TRNrv-Trial 1, n = 11 neurons, two-tailed one-sample t-test, t = 1.242, P = 0.243; TRNrv-Trial 2, n = 10 neurons, two-tailed one-sample t-test, t = 0.697, P = 0.503) or TRNrd neurons (TRNrd-Trial 1, n = 4 neurons, two-tailed one-sample t-test, t = 0.261, P = 0.811; TRNrd-Trial 2, n = 8 neurons, two-tailed one-sample t-test, t = 1.169, P = 0.281). **e** Left, Responses of 5 sec baseline and first 5 secs of the conditioned tone are compared. Significant increase were observed in TRNrv neurons (TRNrv-Day 2, n = 23, one-sample signed rank test, Z = 3.133, P = 0.002) but not in TRNrd neurons (TRNrd-Day 2, n = 6, two-tailed one-sample t-test, t = 2.312, P = 0.0687). Right, full presentation of normalized firing responses of TRNrv (black line) or TRNrd (gray line) neurons during 30 sec tone in extinction learning. All data are presented as mean ± SEM. N.S., not significant. **P < 0.005. See Supplementary Table 1 for values of post hoc test.

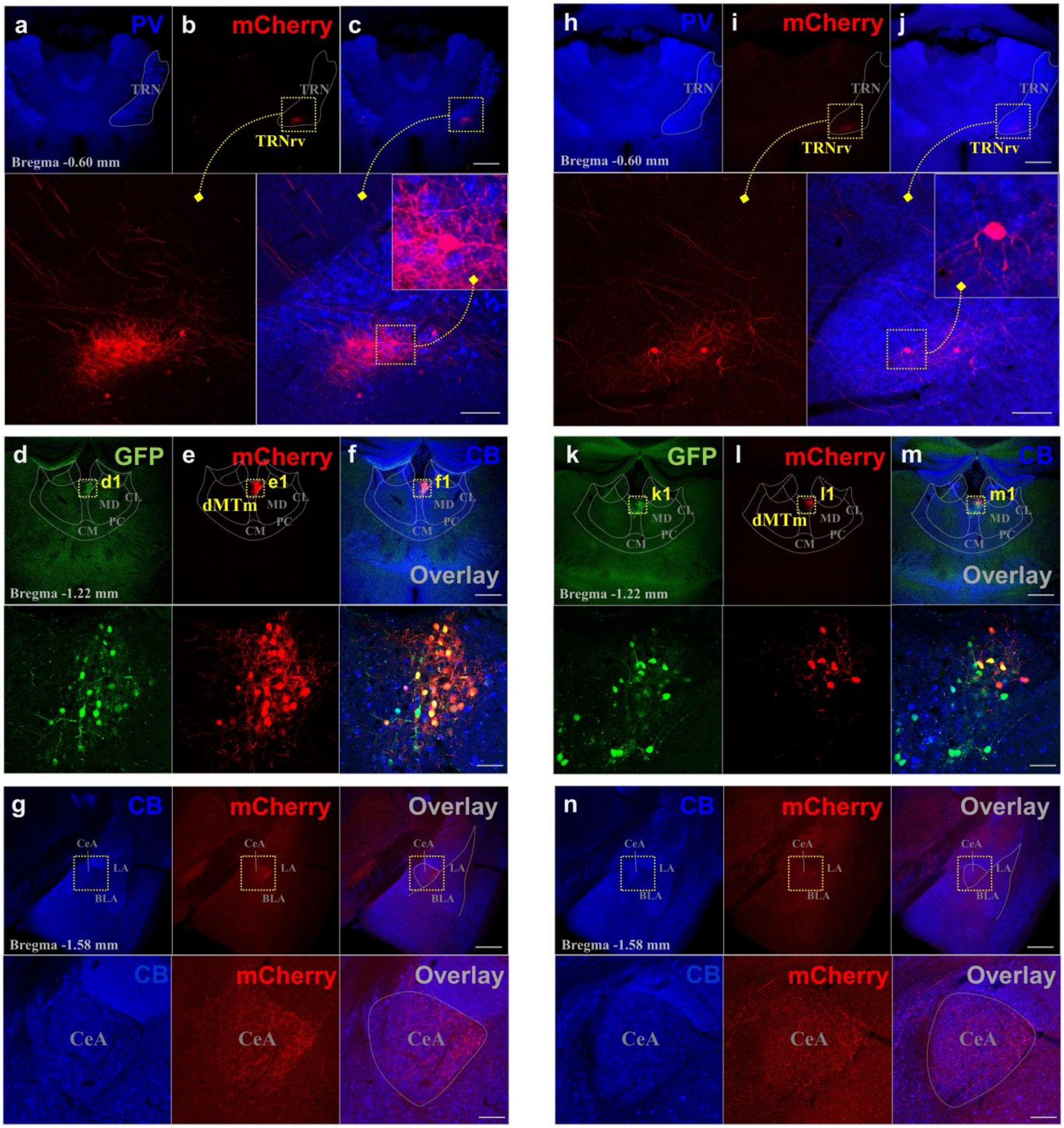

Supplementary Figure 8. Replications of Fig. 6 with two mice. TRNrv neurons are disynaptically connected to the CeA through the dMTm.
a-g Same as Fig. 6a-h. **h-n** Same as Fig. 6a-h.

Supplementary Table

Supplementary Table 1. Related to statistics for experiments shown in Figures and Supplementary Figures

Figure	Experiment Level 1	Comparison Group (sample size)	Experiment Level 2	Measurement	Statistical Test	Statistics (df)	* p<0.05 # p<0.01 ** p<0.005 *** p<=0.001	p-value
Fig. 2d	Condi. (Day1)	CTR.(12) vs. STIM.(13)	3rd tone	Freezing	Mann-Whitney Rank Sum Test	U = 75.0		0.892
Fig. 2d	Extinct. (Day2)	CTR.(12) vs. STIM.(13)	1st tone	Freezing	two-tailed t-test	$t_{(23)}$ = -0.147		0.884
Fig. 2d	Extinct. (Day2)	CTR.(12) vs. STIM.(13)	2nd~18th tones	Freezing	two-way RM-ANOVA (main effect of group)	$F_{(1,23)}$ = 21.555	***	< 0.001
					(main effect of trial)	$F_{(16,368)}$ = 0.663		0.83
					(group x trial interaction)	$F_{(16,368)}$ = 0.756		0.735
Fig. 2d	Extinct. (Day2)	CTR.(12) vs. STIM.(13)	2nd tone	Freezing	two-way RM-ANOVA, Holm-Sidak multiple comparisons	t = 0.188		0.851
			3rd tone			t = 1.285		0.2
			4th tone			t = 1.682		0.094
			5th tone			t = 2.56	*	0.011
			6th tone			t = 2.541	*	0.012
			7th tone			t = 3.271	***	0.001
			8th tone			t = 2.409	*	0.017
			9th tone			t = 1.803		0.073
			10th tone			t = 2.001	*	0.046
			11th tone			t = 2.181	*	0.03
			12th tone			t = 2.336	*	0.02
			13th tone			t = 1.585		0.114
			14th tone			t = 2.326	*	0.021
			15th tone			t = 3.097	**	0.002
			16th tone			t = 2.694	#	0.007
			17th tone			t = 1.799		0.073
			18th tone			t = 2.661	#	0.008
Fig. 2d	Retrieval Test (Day3)	CTR.(12) vs. STIM.(13)	1st~4th tones	Freezing	two-way RM-ANOVA (main effect of group)	$F_{(1,23)}$ = 54.679	***	< 0.001
					(main effect of trial)	$F_{(3,69)}$ = 3.078	*	0.033
					(group x trial interaction)	$F_{(3,69)}$ = 1.130		0.343
Fig. 2d	Retrieval Test (Day3)	CTR.(12) vs. STIM.(13)	1st tone	Freezing	two-way RM-ANOVA, Holm-Sidak multiple comparisons	t = 5.619	***	< 0.001
			2nd tone			t = 5.356	***	< 0.001
			3rd tone			t = 3.538	***	< 0.001
			4th tone			t = 4.502	***	< 0.001
Fig. 2f	Condi. (Day1)	CTR.(12) vs. STIM.(15)	3rd tone	Freezing	two-tailed t-test	$t_{(25)}$ = -0.268		0.791
Fig. 2f	Extinct. (Day2)	CTR.(12) vs. STIM.(15)	1st tone	Freezing	Mann-Whitney	U = 82.00		0.714

					Rank Sum Test			
Fig. 2f	Extinct. (Day2)	CTR.(12) vs. STIM.(15)	2nd~18th tones	Freezing	two-way RM-ANOVA (main effect of group)	$F_{(1,25)}$ = 6.055	*	0.021
					(main effect of trial)	$F_{(16,400)}$ = 2.414	**	0.002
					(group x trial interaction)	$F_{(16,400)}$ = 0.974		0.484
Fig. 2f	Extinct. (Day2)	CTR.(12) vs. STIM.(15)	2nd tone	Freezing	two-way RM-ANOVA, Holm-Sidak multiple comparisons	t = 0.584		0.56
			3rd tone			t = 0.345		0.73
			4th tone			t = 1.178		0.24
			5th tone			t = 2.315	*	0.021
			6th tone			t = 0.0764		0.939
			7th tone			t = 0.442		0.658
			8th tone			t = 1.073		0.284
			9th tone			t = 2.177	*	0.03
			10th tone			t = 2.615	#	0.009
			11th tone			t = 1.961		0.051
			12th tone			t = 7.368e-16		1
			13th tone			t = 0.655		0.513
			14th tone			t = 1.404		0.161
			15th tone			t = 0.238		0.812
			16th tone			t = 2.006	*	0.046
			17th tone			t = 1.104		0.27
			18th tone			t = 1.541		0.124
Fig. 2f	Retrieval Test (Day3)	CTR.(12) vs. STIM.(15)	1st~4th tones	Freezing	two-way RM-ANOVA (main effect of group)	$F_{(1,25)}$ = 10.442	***	0.003
					(main effect of trial)	$F_{(3,75)}$ = 3.219	*	0.027
					(group x trial interaction)	$F_{(3,75)}$ = 1.233		0.304
Fig. 2f	Retrieval Test (Day3)	CTR.(12) vs. STIM.(15)	1st tone	Freezing	two-way RM-ANOVA, Holm-Sidak multiple comparisons	t = 3.464	***	< 0.001
			2nd tone			t = 1.472		0.146
			3rd tone			t = 2.669	*	0.01
			4th tone			t = 2.181	*	0.033
Fig. 4c	Condi. (Day1)	CTR.(14) vs. STIM.(13)	3rd tone	Freezing	two-tailed t-test	$t_{(25)}$ = -0.404		0.69
Fig. 4c	Extinct. (Day2)	CTR.(14) vs. STIM.(13)	1st tone	Freezing	two-tailed t-test	$t_{(25)}$ = -0.0832		0.934
Fig. 4c	Extinct. (Day2)	CTR.(14) vs. STIM.(13)	2nd~18th tones	Freezing	two-way RM-ANOVA	$F_{(1,25)}$ = 0.334		0.568
Fig. 4c	Retrieval Test (Day3)	CTR.(14) vs. STIM.(13)	1st~4th tones	Freezing	two-way RM-ANOVA (main effect of group)	$F_{(1,25)}$ = 6.550	*	0.017
					(main effect of trial)	$F_{(3,75)}$ = 0.209		0.89
					(group x trial interaction)	$F_{(3,75)}$ = 1.314		0.276
Fig. 4c	Retrieval Test	CTR.(14) vs. STIM.(13)	1st tone	Freezing	two-way RM-	t = 2.016	*	0.048

	(Day3)		2nd tone		ANOVA, Holm-Sidak multiple comparisons	t = 1.298		0.199
			3rd tone			t = 1.191		0.238
			4th tone			t = 3.112	**	0.003
Fig. 5c	Condi. (Day1)	Unstim.(13) vs. EYFP (10) vs. NpHR (11)	5th tone	Freezing	Kruskal-Wallis one-way ANOVA on Ranks	$H_{(2)}$ = 0.576		0.75
Fig. 5c	Extinct. (Day2)	Unstim.(13) vs. EYFP (10) vs. NpHR (11)	baseline	Freezing	Kruskal-Wallis one-way ANOVA on Ranks	$H_{(2)}$ = 3.481		0.175
Fig. 5c	Extinct. (Day2)	Unstim.(13) vs. EYFP (10) vs. NpHR (11)	1st block	Freezing	one-way ANOVA	$F_{(2)}$ = 0.0519		0.95
Fig. 5c	Extinct. (Day2)	Unstim.(13) vs. EYFP (10) vs. NpHR (11)	2nd~10th blocks	Freezing	two-way RM-ANOVA (main effect of group)	$F_{(2,31)}$ = 0.423		0.659
					(main effect of trial)	$F_{(8,248)}$ = 4.003	***	< 0.001
					(group x trial interaction)	$F_{(8,248)}$ = 0.792		0.694
Fig. 5c	Extinct. (Day2)	Unstim.(13) vs. NpHR (11)	2nd~10th blocks	Freezing	two-way RM ANOVA, Holm-Sidak multiple comparisons	t =0.905		0.753
Fig. 5c	Extinct. (Day2)	Unstim.(13) vs. NpHR (11)	2nd block	Freezing	two-way RM ANOVA, Holm-Sidak multiple comparisons	t = 0.169		0.866
			3rd block			t = 0.263		0.991
			4th block			t = 0.474		0.952
			5th block			t = 1.838		0.192
			6th block			t = 0.765		0.83
			7th block			t = 0.11		0.912
			8th block			t = 0.0293		0.977
			9th block			t = 0.603		0.796
			10th block			t = 2.242		0.079
Fig. 5c	Extinct. (Day2)	EYFP (10) vs. NpHR (11)	2nd~10th blocks	Freezing	two-way RM ANOVA, Holm-Sidak multiple comparisons	t = 0.602		0.799
Fig. 5c	Extinct. (Day2)	EYFP (10) vs. NpHR (11)	2nd block	Freezing	two-way RM ANOVA, Holm-Sidak multiple comparisons	t = 0.27		0.955
			3rd block			t = 0.135		0.988
			4th block			t = 0.00554		0.996
			5th block			t = 1.446		0.279
			6th block			t = 0.617		0.787
			7th block			t = 0.326		0.935
			8th block			t = 0.262		0.991
			9th block			t = 0.82		0.799

			10th block			t = 1.249		0.382
Fig. 5c	Extinct. (Day2)	Unstim.(13) vs. EYFP (10)	2nd~10th blocks	Freezing	two-way RM ANOVA, Holm-Sidak multiple comparisons	t = 0.256		0.8
Fig. 5c	Extinct. (Day2)	Unstim.(13) vs. EYFP (10)	2nd block	Freezing	two-way RM ANOVA, Holm-Sidak multiple comparisons	t = 0.445		0.96
			3rd block			t = 0.115		0.908
			4th block			t = 0.468		0.871
			5th block			t = 0.289		0.773
			6th block			t = 0.104		0.917
			7th block			t = 0.446		0.96
			8th block			t = 0.243		0.963
			9th block			t = 0.265		0.792
			10th block			t = 0.886		0.378
Fig. 5c	Retrieval Test (Day3)	Unstim.(13) vs. EYFP (10) vs. NpHR (11)	baseline	Freezing	Kruskal-Wallis one-way ANOVA on Ranks	$H_{(2)}$ = 2.756		0.252
Fig. 5c	Retrieval Test (Day3)	Unstim.(13) vs. EYFP (10) vs. NpHR (11)	1st~2nd blocks	Freezing	two-way RM-ANOVA (main effect of group)	$F_{(2,31)}$ = 5.214	*	0.011
					(main effect of trial)	$F_{(1,31)}$ = 8.123	#	0.008
					(group x trial interaction)	$F_{(1,31)}$ = 1.043		0.365
Fig. 5c	Retrieval Test (Day3)	Unstim.(13) vs. NpHR (11)	1st~2nd blocks	Freezing	two-way RM ANOVA, Holm-Sidak multiple comparisons	t = 2.860	*	0.022
Fig. 5c	Retrieval Test (Day3)	Unstim.(13) vs. NpHR (11)	1st block	Freezing	two-way RM ANOVA, Holm-Sidak multiple comparisons	t = 2.919	*	0.016
			2nd block			t = 2.163		0.07
Fig. 5c	Retrieval Test (Day3)	EYFP (10) vs. NpHR (11)	1st~2nd blocks	Freezing	two-way RM ANOVA, Holm-Sidak multiple comparisons	t = 2.744	*	0.02
Fig. 5c	Retrieval Test (Day3)	EYFP (10) vs. NpHR (11)	1st block	Freezing	two-way RM ANOVA, Holm-Sidak multiple comparisons	t = 2.161		0.07
			2nd block			t = 2.715	*	0.028
Fig. 5c	Retrieval Test (Day3)	Unstim.(13) vs. EYFP (10)	1st~2nd blocks	Freezing	two-way RM ANOVA, Holm-Sidak multiple comparisons	t = 0.0655		0.948
Fig. 5c	Retrieval Test (Day3)	Unstim.(13) vs. EYFP (10)	1st block	Freezing	two-way RM ANOVA, Holm-Sidak multiple comparisons	t = 0.598		0.553
			2nd block			t = 0.714		0.479
Supplementary Fig. 2b	Condi. (Day1)	0.3mA (9) vs. 0.7mA (10)	1st~3rd tones	Freezing	two-way RM-ANOVA (main effect of group)	$F_{(1,17)}$ = 2.089		0.167
					(main effect of trial)	$F_{(2,34)}$ = 93.271	***	< 0.001

					(group x trial interaction)	$F_{(2,34)}$ =5.540	#	0.008
Supplementary Fig. 2b	Condi. (Day1)	0.3mA (9) vs. 0.7mA (10)	1st tone	Freezing	two-way RM-ANOVA, Holm-Sidak multiple comparisons	t = 0.519		0.607
			2nd tone			t = 2.376	*	0.024
			3rd tone			t = 1.813		0.08
Supplementary Fig. 2b	Extinct. (Day2)	0.3mA (9) vs. 0.7mA (10)	baseline	Freezing	two-tailed t-test	$t_{(17)}$ = -0.365		0.719
Supplementary Fig. 2b	Extinct. (Day2)	0.3mA (9) vs. 0.7mA (10)	1st~9th blocks	Freezing	two-way RM-ANOVA (main effect of group)	$F_{(1,17)}$ = 16.815	***	< 0.001
					(main effect of trial)	$F_{(8,136)}$ = 7.537	***	< 0.001
					(group x trial interaction)	$F_{(8,136)}$ = 0.300		0.965
Supplementary Fig. 2b	Extinct. (Day2)	0.3mA (9) vs. 0.7mA (10)	1st block	Freezing	two-way RM-ANOVA, Holm-Sidak multiple comparisons	t = 2.864	#	0.006
			2nd block			t = 3.223	**	0.002
			3rd block			t = 2.553	*	0.013
			4th block			t = 2.814	#	0.007
			5th block			t = 2.785	#	0.007
			6th block			t = 3.278	**	0.002
			7th block			t = 3.837	***	< 0.001
			8th block			t = 2.654	*	0.01
			9th block			t = 2.823	#	0.007
Supplementary Fig. 2b	Retrieval Test (Day3)	0.3mA (9) vs. 0.7mA (10)	baseline	Freezing	Mann-Whitney Rank Sum Test	U = 28.5		0.19
Supplementary Fig. 2b	Retrieval Test (Day3)	0.3mA (9) vs. 0.7mA (10)	1st~2nd blocks	Freezing	two-way RM-ANOVA (main effect of group)	$F_{(1,17)}$ = 9.654	#	0.006
					(main effect of trial)	$F_{(1,17)}$ = 3.343		0.085
					(group x trial interaction)	$F_{(1,17)}$ = 0.0035		0.954
Supplementary Fig. 2b	Retrieval Test (Day3)	0.3mA (9) vs. 0.7mA (10)	1st block	Freezing	two-way RM-ANOVA, Holm-Sidak multiple comparisons	t = 2.852	#	0.009
			2nd block			t = 2.803	*	0.01
Supplementary Fig. 4b	Condi. (Day1)	CTR.(7) vs. STIM.(7)	1st~3rd tones	Freezing	two-way RM-ANOVA (main effect of group)	$F_{(1,12)}$ = 0.0387		0.847
					(main effect of trial)	$F_{(2,24)}$ = 43.768	***	< 0.001
					(group x trial interaction)	$F_{(2,24)}$ = 0.141		0.869
Supplementary Fig. 4b	Condi. (Day1)	CTR.(7) vs. STIM.(7)	1st tone	Freezing	two-way RM-ANOVA, Holm-Sidak multiple comparisons	t = 0.313		0.756
			2nd tone			t = 0.274		0.786
			3rd tone			t = 0.346		0.731
Supplementary Fig. 4b	Extinct. (Day2)	CTR.(7) vs. STIM.(7)	baseline	Freezing	two-tailed t-test	$t_{(12)}$ = 0.113		0.912
Supplementary Fig. 4b	Extinct. (Day2)	CTR.(7) vs. STIM.(7)	1st tone	Freezing	Mann-Whitney Rank Sum Test	U = 23.0		0.902

Supplementary Fig. 5b	Condi. (Day1)	CTR.(11) vs. 10Hz (10) vs. 1Hz (12)	3rd tone	Freezing	one-way ANOVA	$F_{(2)}$ = 1.338		0.278
Supplementary Fig. 5b	Extinct. (Day2)	CTR.(11) vs. 10Hz (10) vs. 1Hz (12)	1st tone	Freezing	Kruskal-Wallis one-way ANOVA on Ranks	$H_{(2)}$ = 0.0246		0.988
Supplementary Fig. 5b	Extinct. (Day2)	CTR.(11) vs. 10Hz (10) vs. 1Hz (12)	2nd~18th tones	Freezing	two-way RM-ANOVA (main effect of group)	$F_{(2,30)}$ = 5.772	#	0.008
					(main effect of trial)	$F_{(16,480)}$ = 1.985	*	0.013
					(group x trial interaction)	$F_{(16,480)}$ = 1.149		0.267
Supplementary Fig. 5b	Extinct. (Day2)	CTR.(11) vs. 10Hz (10)	2nd~18th tones	Freezing	two-way RM ANOVA, Holm-Sidak multiple comparisons	t = 3.062	*	0.014
Supplementary Fig. 5b	Extinct. (Day2)	CTR.(11) vs. 10Hz (10)	2nd tone	Freezing	two-way RM ANOVA, Holm-Sidak multiple comparisons	t = 3.455	**	0.002
			3rd tone			t = 2.634	*	0.028
			4th tone			t = 1.409		0.41
			5th tone			t = 2.856	*	0.015
			6th tone			t = 1.384		0.309
			7th tone			t = 2.449	*	0.031
			8th tone			t = 1.845		0.13
			9th tone			t = 1.208		0.229
			10th tone			t = 2.156		0.065
			11th tone			t = 2.11		0.106
			12th tone			t = 2.45	*	0.046
			13th tone			t = 1.993		0.094
			14th tone			t = 2.119		0.104
			15th tone			t = 2.117		0.105
			16th tone			t = 1.569		0.224
			17th tone			t = 1.687		0.179
			18th tone			t = 2.111		0.072
Supplementary Fig. 5b	Extinct. (Day2)	CTR.(11) vs. 1Hz (12)	2nd~18th tones	Freezing	two-way RM ANOVA, Holm-Sidak multiple comparisons	t = 0.250		0.804
Supplementary Fig. 5b	Extinct. (Day2)	CTR.(11) vs. 1Hz (12)	2nd tone	Freezing	two-way RM ANOVA, Holm-Sidak multiple comparisons	t = 1.997		0.094
			3rd tone			t = 1.915		0.112
			4th tone			t = 0.744		0.706
			5th tone			t = 1.88		0.121
			6th tone			t = 0.112		0.911
			7th tone			t = 0.475		0.636

			8th tone			t = 0.951		0.344
			9th tone			t = 1.224		0.396
			10th tone			t = 0.676		0.501
			11th tone			t = 0.567		0.572
			12th tone			t = 0.247		0.806
			13th tone			t = 0.459		0.647
			14th tone			t = 0.667		0.506
			15th tone			t = 0.62		0.537
			16th tone			t = 0.786		0.434
			17th tone			t = 0.901		0.37
			18th tone			t = 0.147		0.884
Supplementary Fig. 5b	Extinct. (Day2)	10Hz (10) vs. 1Hz (12)	2nd~18th tones	Freezing	two-way RM ANOVA, Holm-Sidak multiple comparisons	t = 2.881	*	0.014
Supplementary Fig. 5b	Extinct. (Day2)	10Hz (10) vs. 1Hz (12)	2nd tone	Freezing	two-way RM ANOVA, Holm-Sidak multiple comparisons	t = 1.58		0.117
			3rd tone			t = 0.82		0.414
			4th tone			t = 0.712		0.478
			5th tone			t = 1.082		0.281
			6th tone			t = 1.521		0.343
			7th tone			t = 2.962	*	0.011
			8th tone			t = 2.809	*	0.017
			9th tone			t = 2.426	*	0.049
			10th tone			t = 2.859	*	0.015
			11th tone			t = 1.601		0.211
			12th tone			t = 2.26		0.05
			13th tone			t = 2.481	*	0.043
			14th tone			t = 1.512		0.248
			15th tone			t = 1.556		0.229
			16th tone			t = 2.367		0.057
			17th tone			t = 2.599	*	0.031
			18th tone			t = 2.297		0.068
Supplementary Fig. 5b	Retrieval Test (Day3)	CTR.(11) vs. 10Hz (10)	1st~4th tones	Freezing	two-way RM-ANOVA, Student-Newman-Keuls multiple comparisons	$q_{(2)}$ = 3.234	*	0.03
Supplementary Fig. 5b	Retrieval Test (Day3)	CTR.(11) vs. 10Hz (10)	1st tone	Freezing	two-way RM-ANOVA, Student-Newman-Keuls multiple comparisons	q = 1.006		0.479
			2nd tone			q = 4.204	**	0.004
			3rd tone			q = 2.786		0.053
			4th tone			q = 2.087		0.145

Supplementary Fig. 5b	Retrieval Test (Day3)	CTR.(11) vs. 1Hz (12)	1st~4th tones	Freezing	two-way RM-ANOVA, Student-Newman-Keuls multiple comparisons	$q_{(2)}$ = 1.395		0.332
Supplementary Fig. 5b	Retrieval Test (Day3)	CTR.(11) vs. 1Hz (12)	1st tone	Freezing	two-way RM-ANOVA, Student-Newman-Keuls multiple comparisons	q = 2.679		0.062
			2nd tone			q = 0.523		0.713
			3rd tone			q = 0.887		0.532
			4th tone			q = 0.258		0.856
Supplementary Fig. 5b	Retrieval Test (Day3)	10Hz (10) vs. 1Hz (12)	1st~4th tones	Freezing	two-way RM-ANOVA, Student-Newman-Keuls multiple comparisons	$q_{(2)}$ = 4.660	#	0.007
Supplementary Fig. 5b	Retrieval Test (Day3)	10Hz (10) vs. 1Hz (12)	1st tone	Freezing	two-way RM-ANOVA, Student-Newman-Keuls multiple comparisons	q = 3.638	*	0.032
			2nd tone			q = 4.8	**	0.003
			3rd tone			q = 3.709	*	0.029
			4th tone			q = 2.381		0.218
Supplementary Fig. 5c	Open Field Test	CTR.(12) vs. 1Hz.(11) vs. 10Hz.(11)	~	Distance	two-way RM-ANOVA	$F_{(2,31)}$ = 0.118		0.889
Supplementary Fig. 5d	Elevated Plus Maze	CTR.(11) vs. 1Hz.(12) vs. 10Hz.(10)	~	Ratio of light/dark arm entries	one-way ANOVA	$F_{(2)}$ = 1.816		0.18
