# Supplementary material for: The rostroventral part of the thalamic reticular nucleus modulates fear extinction
Source: Nat Commun. 2019 Oct 11;10:4637. doi: 10.1038/s41467-019-12496-9 (PMC6789150; doi:10.1038/s41467-019-12496-9)
Supplement: Supplementary file 4 — Description of Additional Supplementary Files [file 41467_2019_12496_MOESM4_ESM.pdf]

Description of Additional Supplementary Files

File Name: Supplementary Movie 1

Description: Related to Fig. 2d. Enhanced fear extinction by optogenetic excitation of the TRNrv. Freezing behaviors of the control group and the stimulated group during extinction learning (Day 2; 1st, 3rd and last tone) and retrieval test (Day 3; 1st tone) are shown.
